# Telehealth Rehabilitation for Cognitive Impairment: Randomized Controlled Feasibility Trial

**DOI:** 10.2196/resprot.9420

**Published:** 2018-02-08

**Authors:** Rachel L Burton, Megan E O'Connell

**Affiliations:** ^1^ Department of Psychology University of Saskatchewan Saskatoon, SK Canada

**Keywords:** cognitive rehabilitation, Alzheimer disease, dementia, telehealth

## Abstract

**Background:**

Nonpharmacological interventions are needed to support the function of older adults struggling with subjective cognitive impairment (SCI), mild cognitive impairment (MCI), and dementia due to Alzheimer disease (AD). Telerehabilitation aims to provide rehabilitation at a distance, but cognitive rehabilitation by videoconferencing has not been explored.

**Objective:**

The objective of this study was to compare goal-oriented cognitive rehabilitation delivered in-person with videoconferencing to determine whether telehealth cognitive rehabilitation appears feasible.

**Methods:**

Random assignment to in-person or telehealth videoconferencing cognitive rehabilitation with a combined between-subjects, multiple baseline single-case experimental design, cognitive rehabilitation was delivered by a therapist to 6 participants with SCI (n=4), MCI (n=1), or dementia due to AD (n=1).

**Results:**

Two of the 6 participants randomly assigned to the telehealth condition withdrew before beginning the intervention. For those who participated in the intervention, 6 out of 6 goals measured with the Canadian Occupational Performance Measure improved for those in the in-person group, and 7 out of 9 goals improved for those in the telehealth group.

**Conclusions:**

Delivery of cognitive rehabilitation by telehealth appeared feasible but required modifications such as greater reliance on caregivers and clients for manipulating materials.

## Introduction

### Background

Populations are aging worldwide [1]. In Canada, the rural population is older and is disproportionally aging relative to the urban population [2]. The incidence of dementia increases with age, and formal dementia services are the least accessible in rural and remote communities where the proportion of older adults is the greatest [2,3]. Long travel distances and transportation difficulties further limit accessibility [3]. Telemedicine, or telehealth, is the remote delivery of health care services, where distance is a critical factor, by means of information and communications technology [1]. Expanding telehealth services has been suggested to reduce disparities in urban and rural health care [4]. Interventions to support the function of older adults with cognitive concerns, including dementia, are needed, and it is essential that these interventions are accessible to all families. Cognitive rehabilitation is a promising but understudied nonpharmacological individualized treatment that has been shown to help individuals with mild cognitive impairment (MCI), early stage dementia due to Alzheimer disease (AD), and vascular dementia set [5] and attain personally important functional goals [6-8]. This study investigated the feasibility and acceptability of delivering cognitive rehabilitation to individuals with subjective cognitive impairment (SCI), MCI, and early stage dementia using telehealth videoconferencing.

### Telemedicine and Dementia

Research on telemedicine and dementia has primarily focused on diagnosis (eg, [9,10]), clinical consultation, follow-up appointments [11], and support for family caregivers [12-14]. In comparison, relatively little work has studied interventions for individuals diagnosed with dementia, although research in this area has begun to emerge. For example, Dal Bello-Haas et al [15] demonstrated that videoconferencing is a feasible method to deliver an exercise intervention for rural individuals with dementia due to AD. These examples of successfully delivering remote interventions for individuals with dementia suggest that remote cognitive rehabilitation could be feasible.

### Telerehabilitation

Telerehabilitation is “the set of instruments and protocols aimed at providing rehabilitation at a distance” [16]. Telerehabilitation has been used to provide a range of interventions to individuals diagnosed with a number of different disorders. For example, telerehabilitation has been used to treat stroke [17], spinal cord injury [18], traumatic brain injury [19,20], multiple sclerosis [21], and cognitive impairment following intensive care [22]. Diverse use of telehealth includes delivering diagnostic assessments, caregiver support groups, individual and group psychotherapy [23], home exercise programs, clinical consultations, and cognitive rehabilitation using information and communications technology [16].

### Cognitive Rehabilitation for Dementia

Clare and colleagues have developed a goal-focused approach to cognitive rehabilitation for individuals with early stage dementia due to AD, or mixed AD, and vascular dementia [7,24]. In this approach, cognitive rehabilitation begins with an assessment, which is followed by collaborative goal setting [24]. Typically, functional goals related to everyday memory problems, practical skills, and activities and concentration are set, and improved function in these areas has been reported in multiple studies (eg, [25-31]). Generally, these collaborative goals are addressed in weekly 1-hour sessions using empirically supported techniques such as spaced retrieval, cuing and fading, errorless learning, and external memory aids [24]. Briefly, errorless learning involves training without allowing trial-and-error guessing; cues and supports are given to maximize the probability of guessing the correct response instead [24]. Numerous methods facilitate errorless learning, which includes cuing, gradual fading of cues, and spaced retrieval where recollection occurs over increasing time intervals [24]. Although promising, research evaluating cognitive rehabilitation for individuals with early stage dementia is still emerging [32].

### Remotely-Delivered Cognitive Rehabilitation

The majority of literature on remotely-delivered cognitive rehabilitation focuses on interventions with individuals who have a traumatic brain injury (TBI). Early research suggests that remotely-delivered rehabilitation for individuals who have sustained TBI is feasible (eg, [33,34]). For example, Tam and colleagues [35] reported a series of 3 case studies where individuals with TBI participated in cognitive rehabilitation using customized software. This software combined videoconferencing with screen-sharing, and participants completed computer-based activities that targeted word recognition, semantic memory (ie, memory for factual knowledge), and prospective memory (ie, memory to perform something in the future). In other work, Bergquist and colleagues [33] opted to use technology to remotely teach participants who had had a severe TBI to use a calendar as a compensatory memory strategy. They adapted Sohlberg and Mateer’s [36] calendar training procedure to an instant messenger format and also taught participants to use a personal diary [33]. Both interventions led to increased use of compensatory strategies and improved mood [33]. Finally, in an approach that is more similar to the type of cognitive rehabilitation reported here, Bourgeois and colleagues [37] had participants with chronic TBI identify three everyday memory problems (ie, forgetting appointments, forgetting day planner at home, and losing items), and they provided either an errorless learning approach, spaced retrieval, or memory strategy instructions over the telephone. Individuals in the spaced retrieval group made greater gains in their target goals than those given strategy instructions, and both groups improved their everyday memory functioning. These studies suggest that traditional, in-person cognitive rehabilitation strategies can be delivered by videoconferencing, instant messaging, or telephone.

Remotely-delivered cognitive rehabilitation has also been demonstrated for persons with dementia. Joltin et al [38] used the telephone to train spaced retrieval, a memory intervention, to help 3 women previously diagnosed with dementia recall target information. The goals addressed using spaced retrieval were set in collaboration with family caregivers, staff at the assisted living facility, and the individual diagnosed with dementia [39]. Two participants set the goal to recall what time to take their medications, and one participant set the goals to recall her grandson’s names, her room number, and the year [39]. The first participant (Mini-Mental State Exam, MMSE=17) did not always answer the telephone when the researchers called to provide spaced retrieval training and after 4 sessions, she was still unable to recall the times to take her medication for longer than 2 min [39]. The second participant (MMSE=17) was able to pick up a prompt card listing the times she needed to take her medications across a 5-min interval at the conclusion of the intervention. The third participant (MMSE=13) achieved all three of her goals (grandchildren’s names, room number, and year) and was able to recall the target information across 3 sessions [39]. The authors concluded that it is feasible to modify spaced retrieval for remote delivery. To date, no research has explored videoconferencing for remote delivery of cognitive rehabilitation.

### Objectives

Telerehabilitation is a developing field with the promise of increasing the accessibility of specialized interventions such as cognitive rehabilitation. To date, remotely-delivered cognitive rehabilitation for persons with dementia has not been systematically studied. Interventions that are included in cognitive rehabilitation, such as spaced retrieval, have been applied in a telerehabilitation format, suggesting that this may be an acceptable and feasible approach to increasing the accessibility of cognitive rehabilitation for dementia for persons residing in rural and remote areas. The purpose of this study was to investigate the acceptability and feasibility of delivering cognitive rehabilitation to individuals diagnosed with dementia due to AD using telehealth videoconferencing.

## Methods

### Experimental Design

This small-scale randomized control trial used a combined single-case and between-subjects design. Random assignment to in-person versus videoconferencing conditions occurred by random number generator. Random assignment to condition occurred before recruitment (conditions were determined before the study commenced and hidden in envelopes) and was not stratified by diagnosis or by rehabilitation goal. Regardless of randomized condition, all participants received an in-person initial assessment. At the initial in-person assessment participants selected at least two goals for cognitive rehabilitation. The features of a between-subjects design were combined with the features of a multiple-baseline design. Multiple baselines were measured within-subjects, and treatment modalities were compared across participants. After 3 weeks of baseline assessment, Goal 1 was targeted and baseline assessment for Goal 2 continued. After 3 weeks of Goal 1 intervention, Goal 2 was targeted. In this way, both the in-person and telehealth groups were observed repeatedly during the baseline and treatment phases. The repeated observations over the baseline and treatment phases meet the criteria for a multiple-baseline design (across groups) [39]. In single-case experimental design guidelines, three is the minimum number of data points required to establish a baseline, and the minimum number of data points needed in each phase [40].

### Participants

The University of Saskatchewan Behavioural Ethics Review Board provided ethical approval. Participants were recruited through community-based organizations and a hospital-based geriatric assessment program. Initially, the researchers hoped to recruit participants solely from clinical settings, but low enrollment led us to expand the recruitment strategy and inclusion criteria. With the expanded criteria, individuals with SCI and no diagnosis, MCI, early stage dementia due to AD, or mixed AD and vascular dementia, were all invited to contact us if they were interested in participating in the study. Diagnosis was self-reported (ie, participants reported that they had received a diagnosis of dementia due to AD from their neurologist, reported no diagnosis), but all self-reported diagnoses were consistent with the clinical interview, neuropsychological tests, and questionnaires administered in the assessment for the study. Prior to enrolling in the study, participants completed a brief clinical interview where cognitive rehabilitation was reviewed, informed consent was obtained (from participant and caregiver wherever appropriate), and a Mini-Mental State Exam (MMSE; [41]) was administered. All individuals were encouraged to participate with a family member or friend, but this was not mandatory.

### Measures

Two sets of measures were used in this study: pre-post measures and weekly measures. A set of measures was administered in-person to all participants before and after the intervention. Second, weekly observational measures and measures of goal performance and satisfaction were collected using the medium of in-person versus telehealth as per the random assignment. The measures were selected to be similar to those used by Clare and colleagues in their 2010 randomized control trial [7].

#### In-Person Initial Assessment and Posttreatment Measures

All participants completed neuropsychological testing and self-report measures of mood, anxiety, and quality of life. Support persons completed measures of quality of life (self and participant), function (participant), and caregiver burden.

##### Rivermead Behavioral Memory Test III (RBMT-III)

The RBMT-III was developed to detect memory impairment and change in memory impairment over time [42] and detect problems that may interfere with rehabilitation [43]. The alternate forms reliability of the subtests of the RBMT-III range from *r*=.58 to *r*=.68 in a mixed clinical sample [44]. The RBMT-III differentiates between individuals with and without brain injury [44], and the RBMT-III’s subtests correlate with other similar cognitive tests, with observations of everyday memory failures, and with subjective ratings of memory performance completed by patients and relatives [44]. There are no RCIs reported in the literature; Wilson et al. [44] reported SEM.

##### Delis Kaplan Executive Function System (D-KEFS), Verbal Fluency Subtest

The verbal fluency subtest of the D-KEFS includes letter fluency, category fluency, and category switching [45]. The letter fluency condition, where individuals are asked to say words that begin with a particular letter has high (.80-.89) internal consistency [43]. The category fluency condition, where individuals are asked to say words from a particular semantic category (eg, boy’s names) has adequate (.70-.79) test-retest reliability. The category switching condition, where individuals are asked to alternate between saying words from two different semantic categories (eg, fruit and furniture) has low (<.59) test-retest reliability. RCI’s have been reported by Brooks et al. [46].

##### Test of Everyday Attention (TEA)

The TEA is a measure of attentional processes, and participants completed elevator counting and elevator counting with distraction subtests [47]. Elevator counting measures sustained attention, and elevator counting with distreaction measures selective attention/working memory [43]. The reliability of the map search, elevator counting, and elevator counting with distraction subtests was adequate (*r*=.75-.86) [43]. The TEA is a theoretically-based test of attention, and, further evidence of its convergent and discriminant validity and its psychometric properties in clinical samples is needed [43]. To measure change, there are no RCIs reported in the literature.

##### Quality of Life in Alzheimer Disease (QoL-AD)

The QoL-AD scale is a 13-item questionnaire completed by both the individual diagnosed with AD and his or her caregiver to generate self and informant ratings [48]. The QoL-AD has adequate internal consistency and test-retest reliability, and there is evidence for its validity as a measure of quality of life in persons with AD [48]. A recent review of measures of health-related quality of life for individuals diagnosed with dementia [49] reported good evidence for QoL-AD’s internal consistency, test-retest reliability, content validity, convergent validity, and responsiveness.

##### World Health Organization Quality of Life Assessment, Short Version (WHOQOL-BREF)

Caregivers completed the WHOQOL-BREF, which is a 26-item questionnaire covering the physical, psychological, social, and environmental aspects of quality of life [50]. The measure had good to excellent reliability and there was preliminary evidence for the measure’s validity [50], and more adequate psychometric properties were found with older adults [51]. Skevington et al. [50] did not provide an overall internal consistency reliability, but instead they reported for each subscale: these were averaged (average reliability .778; ranging from .82 to .68 for the 4 subscales), and SDs were pooled (ranging from 2.6 to 3.2) based on the sample of 11830 to equal 2.88.

##### Zarit Burden Inventory (ZBI)

The ZBI is a self-report measure of caregiver burden and the short form of the ZBI has adequate internal consistency (Cronbach alpha=.88-.90), and there is robust evidence for its predictive validity [52,53].

#### Weekly Measures

Performance and satisfaction ratings on the goals for cognitive rehabilitation were measured with the Canadian Occupational Performance Measure 4th Edition (COPM; [54]). The COPM is a semistructured interview where clients identify problems related to self-care, productivity, and leisure; they rate the importance of each of these identified problems and their satisfaction with each problem from 1 (“not able to do it” or “not satisfied at all”) to 10 (“able to do it very well” or “extremely satisfied”) [55]. The COPM was designed to measure change in performance and satisfaction with performance, it is responsive to change, and a two-point change has been established as clinically significant [56]. The COPM has demonstrated adequate test-retest reliability (.84-.92), and there is evidence for the measure’s content, criterion, and construct validity [54].

The number of learning trials related to a specific goal were observed and recorded. For example, if an individual set the goal to learn the names of the members of a social group or improve recall of personal information this was addressed using vanishing cues and spaced retrieval to reduce errors and be consistent with the principles of errorless learning [24]. The measure was the proportion of items correctly recalled. Or, if an individual set the goal to keep track of the date and the plans for the day, this was addressed using prompting and fading to teach the use of a calendar, and the measure was the level of prompting required.

### Intervention Phase

Cognitive rehabilitation followed the procedures outlined by Clare [24] in her manual *Neuropsychological Rehabilitation and People With Dementia.* This manual emphasizes individualized, person-centered goal setting. One participant set goals related to mood and sleep. Here, cognitive behavioural strategies were used to treat insomnia [57] and low mood [58,59]. All of the interviews, assessments, and interventions were completed by a senior doctoral student in clinical psychology (RLB) and supervised by a neuropsychologist (MEO).

### Procedure

#### Assessment

First, all participants participated in an in-person assessment where the pretreatment testing and an interview were conducted. The assessments were carried out over 1 or 2 sessions, based on the scheduling availability of the participants.

#### Baseline Phase

Following the assessment, goals for cognitive rehabilitation were set collaboratively, and baseline performance and satisfaction was measured using the COPM for all goals during 3 baseline sessions (labeled B1, B2, and B3 on the figures below). Measurement occurred either in-person or through telehealth, depending on the experimental condition. Following 3 weeks of baseline measurement, each participant’s first goal was addressed in the subsequent cognitive rehabilitation sessions. Baseline COPM measurement continued for all goals that were not the target of the intervention (ie, Goal 2 and Goal 3).

#### Intervention Phase

The cognitive rehabilitation intervention followed the guidelines provided by Clare [24] in her text on cognitive rehabilitation for people with dementia. Each participant’s first goal(s) were addressed in cognitive rehabilitation on the fourth week, following the baseline phase. A new goal, or set of goals, was introduced every 3 weeks (ie, in CR 4, and in CR 7).

For all participants, the treatment phase was designed to take place over 8 weeks. This decision was based on the procedure reported in Clare and colleagues [7]. Participants attended the Video Therapy Analysis Lab on the University of Saskatchewan campus once a week for a 1-hour session. The setting was the same regardless of in-person versus telehealth condition, but for the telehealth condition, the therapist was not in the same room and videoconferencing was used.

#### Research Journal

RLB kept a research journal during this study beginning in the recruitment phase. Entries were made in the journal after each assessment, baseline, and intervention session. Journal entries documented what took place in the sessions, reflections on the experience of delivering the intervention, and emphasized any adaptations that were made to make cognitive rehabilitation more amenable to videoconferencing.

### Data Evaluation

#### Evaluation of the Quantitative Data Provided by Participants

The data from the study were evaluated using visual inspection. In single-case research visual inspection is the primary method of data evaluation and, although statistical methods for evaluating single case data are increasingly available, they are not widely used [39]. Visual inspection is based on exploration of changes in the magnitude of the data and changes in the data across phases (eg, from the baseline to the intervention phases). There are two characteristics of single-case data related to magnitude: changes in means across phases and changes in level across phases [39]. A change in means refers to a change in the average of a measure in one phase to another. A change in level refers to a shift, jump, or discontinuity in the data from the end of one phase to the beginning of another. There are also two characteristics related to rate of change: changes in trend and latency. A change in trend is a change in the slope of the data from one phase to the next. A change in latency refers to the period of time that elapses from the time the phase changes (ie, the onset of the intervention) until there is a change in the data.

Visual inspection is a reliable method of data evaluation when the results are strong, and changes from one phase to the next are clear [60]. Therefore, visual inspection encourages researchers to study interventions that have potent effects that are readily observable because weak results are generally not visible under visual inspection [39]. The insensitivity of visual inspection to weak results is often considered to be a strength of this approach rather than a limitation. For example, looking for consistent results that can be easily seen also minimizes the chances of making a Type I error (concluding that the intervention has an effect when the results are because of chance; [39]). In this multiple-baseline study, the researchers were interested in determining whether there was a significant change in performance from the baseline to intervention phase. Changes in level and trend were both of interest.

#### Evaluation of Qualitative Data Provided in the Research Journal

The journal documenting the experience of adapting cognitive rehabilitation to telehealth videoconferencing was analyzed thematically. Journal entries were organized into a descriptive summary based on the method of qualitative description detailed in Sandelowski [61,62], and the technique of thematic analysis was as described by Braun and Clark [63]. The thematic analysis took a theoretical approach (as opposed to an inductive approach) insofar as the researchers specifically coded responses related to ways in which the videoconferencing-delivered intervention needed to be modified. This method of qualitative description is a low inference qualitative methodology, and it is intended to generate a comprehensive summary of an event in everyday terms [61].

## Results

### Participants

Eight individuals were recruited to participate in this study, participants were immediately randomly assigned to in-person versus telehealth, and the initial assessment was completed in-person. Two discontinued the study following the initial assessment, and these two happened to be randomly assigned to the telehealth condition. In one case, the family member support person reported that she and the participant did not have time to participate. In the other case, the family member support person reported that initial assessment had been distressing for the participant, and following a family discussion it had been decided that participating in the study was likely to be more distressing than helpful. Demographic and descriptive data for the six individuals who participated are presented in Tables 1 and 2.

**Table 1:** Participant characteristics.

Participant	Delivery	Age	Years of education	Gender	Recruitment source	Diagnosis	Relationship to support person	Involvement of support person
A	In-person	72	18	Female	Support organization	AD^a^	Husband	Attended all sessions
B	In-person	68	14	Male	Support organization	MCI^b^	Wife	Initial interview and questionnaires
C	Telehealth	80	16	Female	Community	SCI^c^	None available	None
D	Telehealth	66	13	Female	Community	SCI	Husband	Initial interview and questionnaires
E	In-person	77	12	Female	Community	SCI	Husband	Questionnaires only
F	Telehealth	68	16	Female	Community	SCI	Husband	Initial interview and questionnaires

^a^AD: dementia due to Alzheimer disease.

^b^MCI: mild cognitive impairment.

^c^SCI: subjective cognitive impairment.

**Table 2:** Initial assessment (1st) and postcognitive rehabilitation (2nd) assessment measures for participants and support persons. Initial assessment (1st) and postintervention (2nd) measures for participants randomly assigned to the in-person cognitive rehabilitation group. Participants were all encouraged to participate with a support person, but participants C and E stated that no support person was available to participate, consequently missing data exist for caregiver reported items for these participants. "dc" indicates caregiver data are missing (discontinued).

Measure^a^		Maximum SE_D_/RCI^b^	A		B		C		D		E		F	
			In-person		In-person		Telehealth		Telehealth		In-person		Telehealth	
			1st	2nd	1st	2nd	1st	2nd	1st	2nd	1st	2nd	1st	2nd
MMSE		30	17		27		29		27		29		26	
Memory-RBMT-III		194^c^	55	45	101	103	158	151	158	152	152	158	106	144
Memory-RBMT-III		100th percentile, SE_D_=7.6^d^	0.2	0.2	4	4	92	82	93	82	84	92	5	63
**Executive Function-DKEFS**		19^c^												
	Letter fluency	RCI=2.7^e^	6	2	11	12	8	9	13	16	14	14	15	16
	Category fluency	RCI=3.1^e^	3	3	9	5	10	14	18	16	18	18	16	17
	Switching total correct	RCI=5.8^e^	1	1	8	8	14	13	19	18	17	18	15	14
	Switching total switch	RCI=5.4^e^	1	1	10	9	15	13	17	14	17	14	15	13
**Attention-TEA**														
	Elevator count	7 raw^f^	6	4	6	7	7	-	7	7	7	7	7	7
	Elevator distraction	SE_D_=0.8^f^	dc	dc	5	6	dc	-	11	11	13	9	5	6
QoL-AD		52, SE_D_=3.8^g^	25	48	34	34	108	109	106	108	28	-	115	118
ADLs-Bristo		60, SE_D_=4.0^h^	0	1	3.5	1	0	0	0	0	0	0	-	0
Anxiety-HADS		21, MCID=1.4^i^	4	4	8	5	6	0	6	7	10	10	4	2
Depression-HADS		21, MCID=1.6^i^	0	3	2	2	1	1	2	0	9	7	2	0
**Caregiver measures**														
	Quality of life-WHOQOL-BREF	130, SE_D_=1.9^j^	103	79	86	91	-	-	91	96	-	-	126	110
	Quality of Life-AD	52, SE_D_=3.8^g^	40	36	30	22	-	-	0	-	-	-	0	0
	ADLs-Bristol	60, SE_D_=4.0^h^	18	14.5	7.5	2.5	-	-	6	0	-	-	10	0
Caregiver Burden-ZBI		36, SE_D_=7.0^k^	35	-	37	51	-	-	13	-	-	-	6	-

^a^Acronyms: MMSE: Mini-Mental State Exam; RBMT-III: Rivermead Behavioural Memory Test III; DKEFS: Delis Kaplan Executive Function System; TEA: Test of Everyday Attention; QoL-AD: Quality of Life in Alzheimer Disease; ADLs-Bristol: Bristol Activities of Daily Living Scale; HADS: Hospital Anxiety and Depression Scale; MCID: Minimum Clinically Important Difference; WHOQOL-BREF: World Health Organization Quality of Life Assessment, short version; ZBI: Zarit Burden Inventory.

^b^Standard error of the difference (SED) is the SD of the expected test-retest difference score if no change has occurred; accounts for standard error in measurement (SEM) at both time points; SED=square root of 2 times the SEM squared. SEM=SD times square root of 1 – reliability. Reliable change indices (RCI) incorporate SED and expected improvement in performance due to practice effects or expected changes due to standard error in prediction and regression to the mean in addition to practice effects, depending on the RCI formula.

^c^Refers to standard score.

^d^No RCIs reported in the literature; [44] reported SEM.

^e^RCIs from [46]; 90th percentile with average practice effect used.

^f^No RCIs reported in the literature, reliability of elevator counting not reported due to ceiling effect, reliability of elevator counting with distraction [43] reliability .857; SD 1.42.

^g^Internal consistency reliability .82; SD 6.3 [64].

^h^Test-retest reliability=.95; SD 12.7 [65].

^i^MCID: Minimum Clinically Important Difference [66] detail changes in HADS scores that were important based on external measures, which is a suggested method for determining MCID.

^j^[50] did not provide an overall internal consistency reliability, but instead they reported for each subscale: these were averaged (average reliability .778; ranging from .82 to .68 for the 4 subscales), and SDs were pooled (ranging from 2.6 to 3.2) based on the sample of 11830 to equal 2.88.

^k^Internal consistency reliability .90; SD 15.64 [52].

### Goals and Completion

Participants each collaboratively set between one and five goals for cognitive rehabilitation. Table 3 lists the specific goals and the cognitive rehabilitation strategies used to address them. The study was designed to deliver 8 sessions of cognitive rehabilitation after 3 sessions of the baseline phase. All 3 participants randomly assigned to the in-person intervention completed 8 sessions. In the telehealth group, one individual completed 8 sessions, one individual (Ms D) completed 7 sessions, and one individual (Ms F) completed 6 sessions. Ms D reported that she had decided to go on vacation, and therefore the researchers decided to cancel the final telehealth rehabilitation session and complete posttreatment assessment. Ms F only had one goal for cognitive rehabilitation, and she felt it had been accomplished after 6 telehealth cognitive rehabilitation sessions.

### Goal Performance

**Figure 1:**
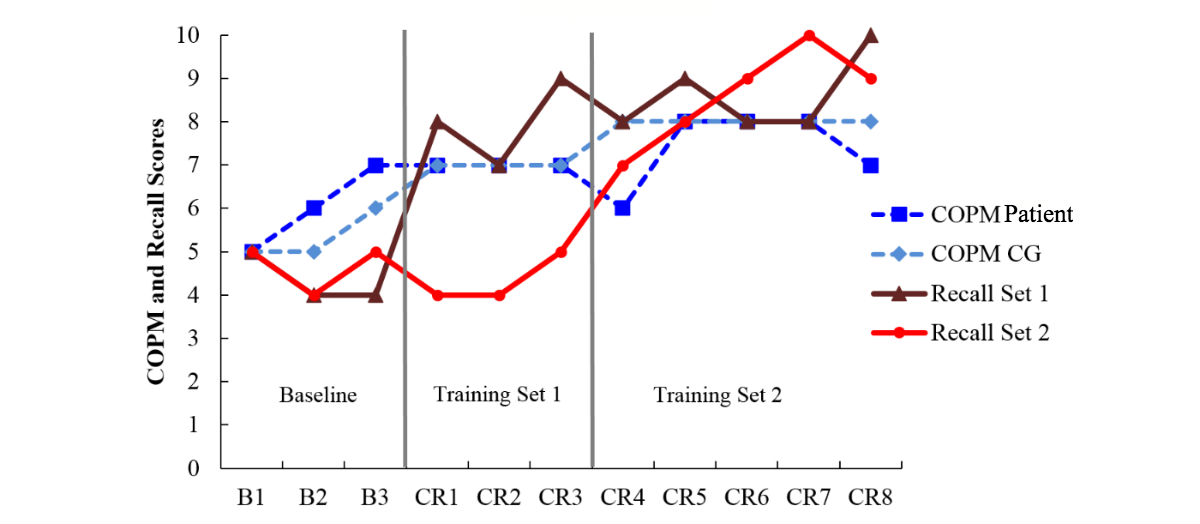
Canadian Occupational Performance Measure (COPM) scores and total item recall scores (two sets of 10) for participant A. The first line indicates when training for Recall Set 1 was initiated and the second line indicates when training for Recall Set 2 was initiated. CG: caregiver; B: baseline; CR: cognitive rehabilitation.

**Figure 2:**
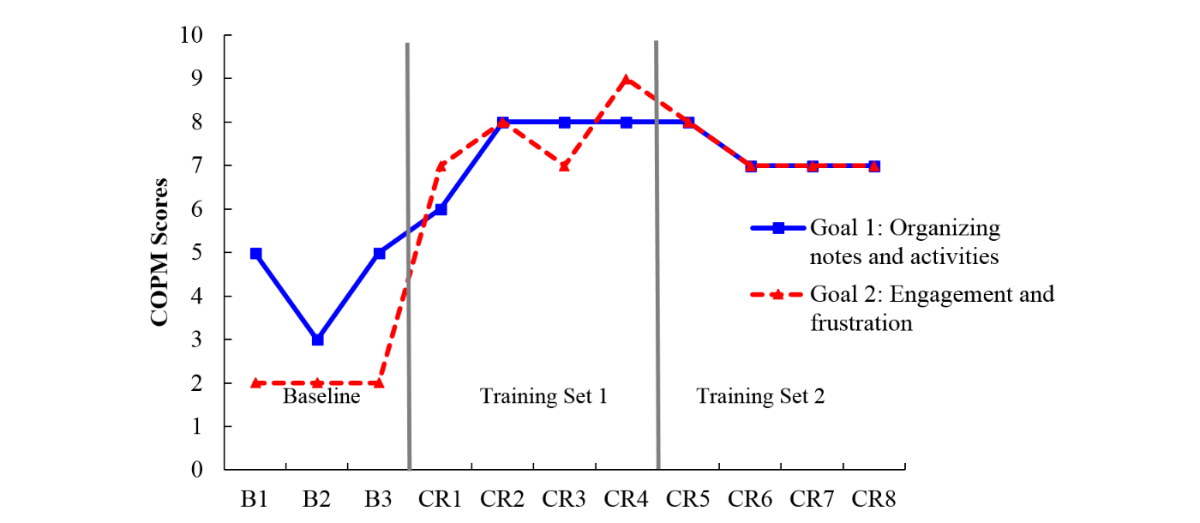
Canadian Occupational Performance Measure (COPM) scores for participant B who attended cognitive rehabilitation sessions in-person. B: baseline; CR: cognitive rehabilitation.

**Figure 3:**
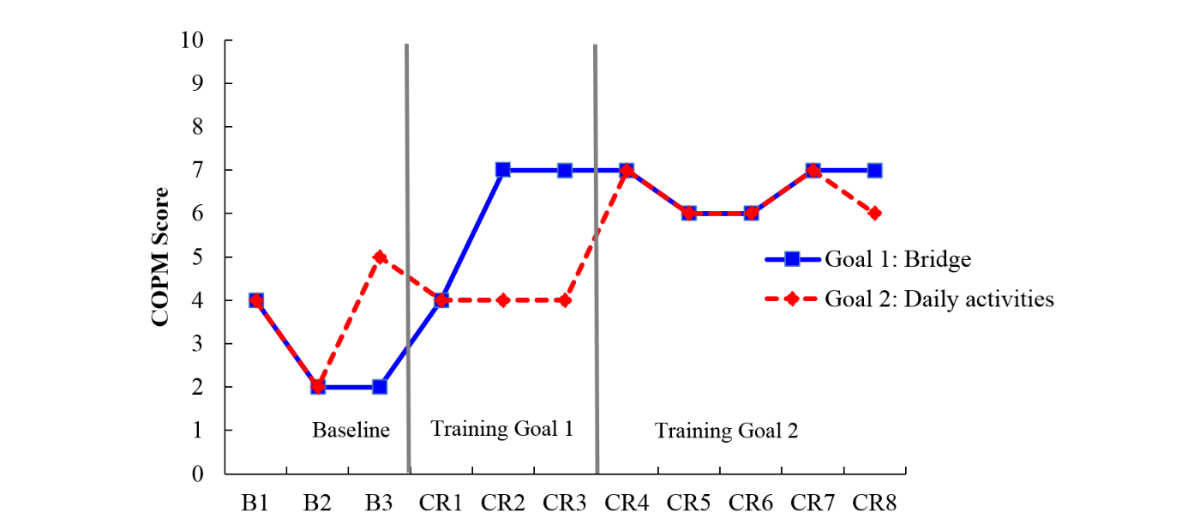
Canadian Occupational Performance Measure (COPM) scores for participant E who attended cognitive rehabilitation sessions through in-person. B: baseline; CR: cognitive rehabilitation.

**Figure 4:**
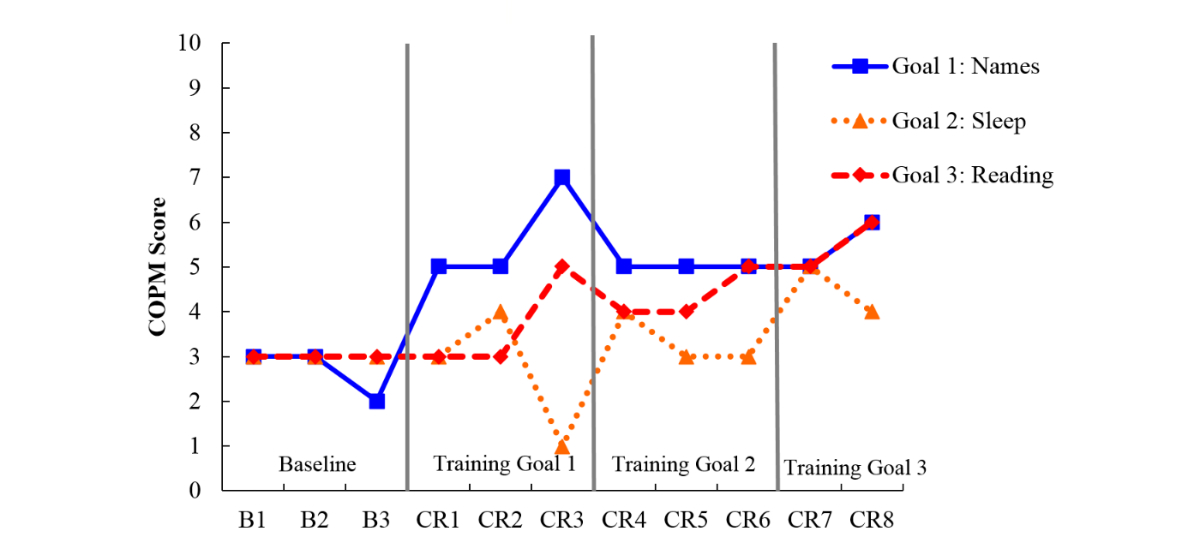
Canadian Occupational Performance Measure (COPM) scores for participant C who attended cognitive rehabilitation sessions through telehealth videoconferencing. B: baseline; CR: cognitive rehabilitation.

**Figure 5:**
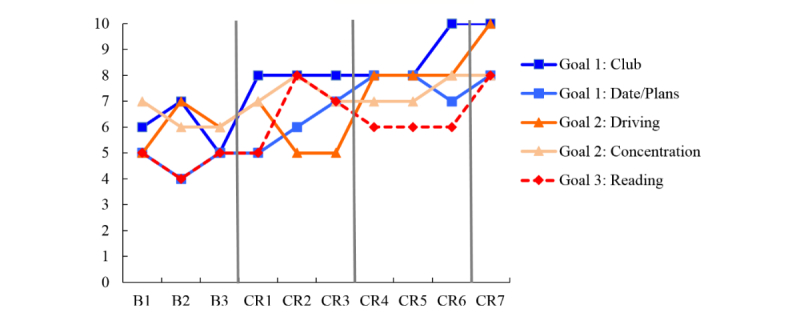
Canadian Occupational Performance Measure (COPM) scores for participant D who attended cognitive rehabilitation sessions through telehealth videoconferencing. B: baseline; CR: cognitive rehabilitation.

**Figure 6:**
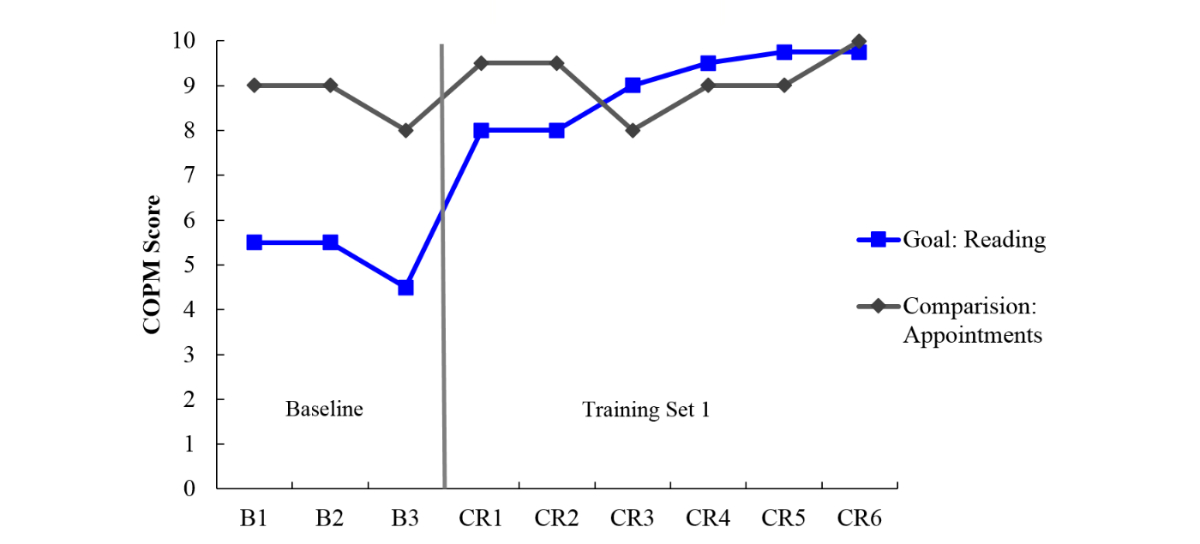
Canadian Occupational Performance Measure (COPM) scores for participant F who attended cognitive rehabilitation sessions through telehealth. Here, only one goal was set to improve recollection when reading. Keeping track of appointments was rated weekly as a comparison measure to provide a second baseline. B: baseline; CR: cognitive rehabilitation.

The primary outcome measure was goal performance as measured by the COPM. Figures 1 to 6 display the COPM scores across the baseline and intervention phases for each of the 6 participants.

**Table 3:** Participants’ goals and cognitive rehabilitation strategies used to address these goals during the intervention.

Participant	Intervention delivery	Goals	Cognitive rehabilitation strategies used to address goals
A	In-person	To remember personally significant life events and accomplishments; To know the names and relationships of important people (eg, grandchildren, siblings, friends).	These two goals were addressed together using an external aid (memory book), which included photos, newspaper clippings, and documents displaying significant people and events. Twenty pages from the memory book were chosen, and these focused on in 2 sets of 10 using spaced retrieval and cuing and fading.
B	In-person	To keep track of date, plans, and activities.	An external aid (day timer) was used to address this goal. Use of the day timer was trained using spaced retrieval and cuing and fading.
		To reduce frustration related to memory and organizational difficulties; feel more engage in activity at hand.	A relaxation exercise and relaxation cues chosen by Mr B were used to address this goal.
C	Telehealth	To recall the names of group members.	Face-name association and spaced retrieval was used to address this goal.
		To improve sleep.	Sleep hygiene, relaxation strategies (eg, deep breathing), and cognitive behavioral (eg, developing alternative thoughts for cognitive distortions) was used to address this goal.
		To remember what was read in a novel or non-fiction book.	External aids and Preview Question Read State Test were used to address this goal.
D	Telehealth	To remember plans and what to bring to club meetings.	External aids (using a single, large day timer), and habits and routines were used to address these goals.
		To keep track of the date and plans for the day.	External aids (using a single, large day timer), and habits and routines were used to address these goals.
		To feel more confident driving and navigating.	Relaxation strategies (eg, deep breathing), external aids (eg, GPS), and habits and routines were used to address this goal.
		To maintain concentration when multitasking at home.	Goal management training was used to address this goal.
		To remember what was read in the newspaper or a novel.	Preview Question Read State Test was used to address this goal.
E	In-person	To remember what was read in bridge books and apply it when playing bridge.	External aids and Preview Question Read State Test were used to address this goal.
		To know what was done from day-to-day and be able to tell friends on the phone.	An external aid (daily journal) and routine was used to address this goal.
F	Telehealth	To keep track of plot and characters when reading a novel.	External aids (eg, notes, sticky tabs, and highlighting) and Preview Question Read State Test were used to address this goal.

#### In-Person Intervention (Participants A, B, and E)

Figures 1,2, and 5 display the session-by-session COPM scores for Ms A, Mr B, and Ms E who were all assigned to participate in cognitive rehabilitation in-person.

Ms A (patient) participated in-person with her husband (caregiver; CG), and their data are represented in Figure 1. Ms A’s goal was to improve her recollection of personally significant life events. A memory book was compiled by Ms A and her husband, and this book was trained using spaced retrieval and fading and cuing in two sets of 10 memory book pages (ie, 2 sets of 10 pages each). Baseline data from all measures was collected. Set 1 was studied in sessions CR 1, 2, and 3 (indicated by the first vertical line in Figure 1). Sets 1 and 2 were both studied in sessions 4, 5, 6, 7, and 8. Visual inspection of Figure 1 suggests Ms A’s (COPM patient) COPM scores were relatively stable across the cognitive rehabilitation, but her husband’s scores increased with the number of intervention sessions. Moreover, Figure 1 demonstrates support for spaced retrieval: recall of both memory book sets was at floor during the baseline, and only recall set 1 improved with the initiation of spaced retrieval (first vertical line in Figure 1) and the untrained set 2 remained at baseline, only improving after initiation of training (second vertical line in Figure 1).

Mr B set two goals for cognitive rehabilitation. First, he wanted to keep better track of his daily notes and musings, which were disorganized. Second, he wanted to reduce feelings of frustration when challenged during a task to feel more engaged in his daily activities (eg, attending club meetings, taking his dog for a walk). Figure 2 shows a moderately stable baseline for Goal 1 and robustly stable initial baseline for Goal 2 as measured by the COPM. At the first intervention session (first vertical line in Figure 2), a consolidated notebook strategy was introduced to target Goal 1, and COPM scores for both goals show a change in level and trend. The change in level is maintained throughout the remainder of the sessions. Although Goal 2 was not explicitly targeted until session CR 4 (the second vertical line in Figure 2) when relaxation techniques and cues were introduced, Goal 2 scores nevertheless appeared to have improved with the intervention targeting Goal 1. Introducing the organizational strategy designed to target Goal 1 had a greater impact on Goal 2 scores than the relaxation exercises designed to address Goal 2. If Mr B’s frustration is conceptualized as being a reaction to cognitive lapses that were not mitigated by his previously disorganized memory aide strategy, this “bleeding” of the organizational intervention from one goal to another goal is expected.

Ms E set two goals for cognitive rehabilitation. Following 3 baseline sessions (B1, 2, and 3) the researchers focused on her goal to improve her recall of bridge (a card game) strategies, which she enjoyed studying. This was addressed using the Preview Question Read State Test [67] strategy, a hierarchical strategy for organizing texts, which was trained using spaced retrieval. Ms E also began to use an external aid (note taking) when reading her bridge books. Visual inspection of Figure 3 shows some variability in the baseline, but consistent and sustained improvement one session after initiation of the intervention aimed at this goal (first vertical line in Figure 3). The baseline sessions for goal 2, keeping track of daily activities, also show variability, but COPM scores clearly increase after this goal was the focus of cognitive rehabilitation (second vertical line in Figure 3).

#### Telehealth Intervention (Participants C, D, and F)

Figures 4 to 6 display the session-by-session COPM scores for Ms A, Mr B, and Ms F, who were all randomly assigned to participate in cognitive rehabilitation through telehealth videoconferencing.

Following 3 baseline assessment sessions Ms C’s cognitive rehabilitation sessions (CR1, 2, and 3; first vertical line in Figure 4) focused first on strategies for learning and remembering names using cuing and fading of face-name associations and spaced retrieval. Next the researchers targeted her sleep (CR 4, 5, and 6; second vertical line in Figure 4) using strategies from cognitive behavioral therapy for insomnia (CBTi), and finally her ability to recall what she read (CR 7, 8; third vertical line in Figure 4) using external aids and the Preview Question Read State Test [67] strategy. Visual inspection of Figure 3 suggests name recall and reading improved, with sleep showing variability through its baseline sessions (B1-3, CR 1-3) and training sessions. Reading performance improved starting at CR 3 suggesting treatment carry over from training naming strategies, which makes sense considering that the strategies for learning and remembering names (ie, face-name associations) require one to slow down, to focus on the information that is being presented, and to work to encode it in a more rich, elaborative manner.

Ms D reported subjective cognitive impairment and set five goals, which were addressed in three training sets (see Table 3). Cognitive rehabilitation was ended after 7 sessions because of a summer vacation for Ms D. Goal set 1 focused on keeping track of day-to-day events, and what to bring to club meetings was addressed using external aids. Ms D was using a number of different systems (cellphone, notebook, day timer), which were consolidated. Visual inspection of Figure 5 suggests that despite some variation in the baseline sessions (B1, 2, and 3), performance on Goal 1 improved by 3 points on the COPM from the highest baseline rating to the highest intervention rating. This increase begins following the first vertical line and is maintained over the course of the remaining sessions. Similarly, performance on Goal set 2, which was concentration and driving, improved when cognitive rehabilitation targeted this goal starting in CR 4 (second vertical line). Goal 3, reading, was targeted only in CR 7 using the Preview Question Read State Test [67] strategy, but performance improved starting in CR 1 and 2, which suggests that the specific training provided during cognitive rehabilitation in CR 7 did not cause the improvements shown in the Figure 5. Rather, the strategies started in CR 1 (external aids) appeared to have supported her goal to recall what she had read. These data suggest that Ms D tended to multitask, and moved quickly from one partially finished task to the next. Using external aids may have reduced the load on her working memory, which would allow her to devote more of her cognitive resources to reading when she picked up a book or newspaper.

Ms F only had one goal for cognitive rehabilitation: she described herself as an avid reader and reported struggling to recall the plot of a novel while reading. Her goal was to be able to keep track of significant characters and their relationships when reading, which was addressed using the Preview Question Read State Test [67] strategy taught through spaced retrieval. This was also combined with external aids including using sticky notes in her books to mark important passages and writing down notes about major characters which she could refer back to. Keeping track of appointments was rated weekly using the COPM as a comparison goal, and served as the second baseline but was never trained. Visual inspection of Figure 6 suggests little variability in this comparison measure, which was, unfortunately likely at ceiling even during the baseline and was therefore never trained. Regarding Ms F’s goal, the baseline phase is stable and substantial improvement in performance is present beginning with cognitive rehabilitation in CR 1 (vertical line in Figure 6).

### Secondary Outcomes

The pretreatment and posttreatment scores for the secondary outcome measures are presented in Table 2. To facilitate comparisons reliable change indices (RCI) were provided where they were available in the literature. When RCI were not available, standard error of the difference (SE_D_) or minimum clinically important differences (MCID) were provided in Table 2.

There were few changes in the secondary measures that exceeded these estimates of change, and only changes greater than the SE_D_, RCI, or MCID are reported below.

#### In-Person Intervention (Participants A, B, and E)

Ms A was the only individual with a dementia due to AD diagnosis who participated in the study, and her scores on the memory measure (RBMT-III) and executive function measure (DKEFS letter fluency) declined in the 12 weeks between the initial assessment and posttreatment assessment. This may reflect disease progress or failure to benefit from practice (the DKEFS RCI includes practice effects). She demonstrated an increase (from 0 to 3) on the Hospital Anxiety and Depression Scale (HADS) depression subscale that was greater than the MCID, but her score remained well below cut-off for clinical depression. Although she reported improved quality of life, her husband reported decreased quality of life over the 12 weeks. The second in-person participant, Mr B had decreased category fluency as measured by the DKEFS, and decreased anxiety as measured by the HADS. His wife reported improved quality of life for herself, decreased quality of life for Mr B, improved function for Mr B, and increased caregiver burden. Finally, Ms E had decreased divided attention as measured by elevator counting with distraction, and decreased depression as measured by the HADS. A support person did not accompany her.

#### Telehealth Intervention (Participants C, D, and F)

Ms C demonstrated improved category fluency and decreased anxiety as measured by the HADS. The second telehealth participant, Ms D, demonstrated improved letter fluency as measured by the DKEFS and decreased depression as measured by the HADS. Her husband reported improved quality of life for himself and improved function for Ms D. Finally, Ms F had improved memory as measured by the RBMT-III and decreased anxiety and depression as measured by the HADS. Her husband reported decreased quality of life for himself and improved function for Ms F.

### Findings From the Research Journal

The research journal was used to reflect on the process of conducting this study, to document any challenges and successes that may not have been fully captured by the quantitative measures, and to document modifications that were made to deliver the intervention to the individuals in the telehealth group. The codes that were generated were organized into two major themes: “relationship and therapeutic alliance” and “method and technique.” Text pertaining to how the researcher (RLB) felt working with the participant, comments the participant made regarding comfort, or how they felt in the session were coded in the “relationship and therapeutic alliance category.” “Engagement” (interest in the intervention and attendance), “connection and enjoyment” (partnership with participants and fun during the sessions), and “responsibility” (researcher’s sense of personal accountability) were coded as subthemes. Text pertaining to study design, measurement, or comparisons between in-person and telehealth treatment were coded in the method and technique theme. “Adjustment to telehealth” and “challenges of measurement” were the themes within “method and technique.” The findings of the thematic analysis are summarized in Table 4, and characteristic examples of text from the journal are presented in the table.

The research journal reflects the importance of building rapport and an alliance to carry out the cognitive rehabilitation. Initial codes were organized into the minor themes of “engagement,” “enjoyment and connection,” and “responsibility.” It is notable that these themes were similar irrespective of treatment modality (in-person vs telehealth).

**Table 4:** Themes from the research journal. Quotes illustrating characteristic examples are observations and reflections made by the researcher (RLB) throughout the study.

Major & minor themes		Characteristic examples
**Relationship and therapeutic alliance**		
	Engagement	*Mr B’s wife explained that she preferred not to attend sessions with her husband because she felt so busy with other commitments. It will be important to have at least one session with her where I show her how we have been using the book.*
		*Mr and Mrs D are both highly engaged. Megan and I discussed the self-selection that is taking place in my recruitment process.*
		*Ms E called me this morning to ask if it would be alright if her husband did not attend. When she arrived she explained the he “doesn’t really believe in mental things” and didn’t think she needed to participate in the study.*
	Connection and enjoyment	*I really enjoyed working with her and found her bright, perceptive, and easily engaged.*
		*He seems to enjoy attending our sessions. Specifically, we laugh and joke a little. He always attends. I am enjoying working with him...some things are a bit challenging/ frustrating. He talks a lot and it can be challenging to interrupt and redirect him to the task at hand.*
		*She is friendly and easy going, and it’s highly enjoyable to work with her.*
		*Ms E commented that participating has been “very interesting and I’ve enjoyed coming.”*
	Responsibility	*This is a deeply personally challenging research project. It is so much more difficult than using archival data because of the personal connection and responsibility I feel towards the research participants.*
		*I have to manage the expectations and the hopes of the participants.*
**Method and technique**		
	Adjustment to telehealth; different but not worse	*She noted that she was disappointed to be assigned to the telehealth videoconferencing condition but would participate.*
		*The volume was too loud and it hurt Ms C’s ears. She easily turned down the volume using the remote control.*
		*I could hear a delay between when I spoke and when my voice played in the testing room which was distracting.*
		*Ms C said it was fine to see and talk to me through videoconferencing. In fact, it was better than expected.*
		*Ms D said it [telehealth] was just fine. Mr D commented that he preferred when we talked face to face and I was in the same room as them. That being said, he agreed with his wife that it was perfectly feasible to work with me through videoconferencing and the goal-setting session had gone well.*
		*There is a bit of overlap in us speaking. Conversing is not quite as natural. Ms F compared it to talking on a cell phone, and not being sure when it was her turn to talk.*
	Greater reliance on verbal description	*I noticed that it was more difficult to see if her chest and abdomen were rising and falling as we practiced diaphragmatic breathing. To compensate, I asked her to describe any spots where she was struggling verbally.*
		*I could not see what was written, so she read what was written to me.*
	Challenge of measurement	*It has been very challenging to balance meeting their goal of developing Ms A’s ability to discuss important autobiographical events with the need to have measureable outcomes.*
		*...it starts to feel “like a test and that’s never fun.”*
		*I have observed marked “spillage” from the intervention items to the baseline items. Ms A now recalls pieces of information about the photos that she could not previously tell me. It will be very difficult to describe whether improvements in Ms A’s descriptions of the pages of her memory book are due to spaced retrieval and prompting and fading, or whether they are due to reminiscence and increased familiarity with the pages in the book.*
		*I am noticing that it is very challenging to address goals purely and there is contamination between goals.*

The theme of “method and technique” is comprised of journal entries that comment on the adaptation of cognitive rehabilitation to telehealth videoconferencing. Participants adjusted easily and quickly to working through telehealth videoconferencing. Journal entries were organized into the subtheme of “different but not worse.” Participants commented that although they might have preferred to meet in-person, the telehealth sessions ran smoothly. As a clinician, the researcher (RLB) noted challenges because of not being able to physically interact with materials. For example, the researcher was unable to pick up a day timer and read through what the participant had written. Therefore, the researcher had to cue participants to read out written notes or from worksheets. Initial codes in the research journal were organized into the minor theme “greater reliance on verbal description.” Lastly, journal entries comment on working to adjust and modify goals and sessions to make the intervention measureable and adhere to the multiple baseline design. Initial codes were organized into the subtheme “measurement challenges.”

## Discussion

### Principal Findings

The results of this study cautiously suggest cognitive rehabilitation can be adapted to telehealth videoconferencing for older adults with subjective and objective memory impairment. The study also adds to the growing body of literature that suggests goal-oriented cognitive rehabilitation delivered in-person is a promising nonpharmacological intervention for older adults with SCI, MCI, and early stage dementia due to AD. For participants who completed the initial assessment and baseline sessions, participation was excellent with all in the in-person group completing 8 out of 8 sessions. Although two in the telehealth group completed fewer than 8 sessions, one terminated because of the goal having been attained and the other terminated because of scheduling conflict with vacation. Both groups demonstrated high completion rates, however the lower rate of session completion for the telehealth group may suggest that telehealth-delivered treatment is less acceptable to participants or something about this modality of treatment (such as the virtual nature of the interpersonal connection, or added challenge of describing steps and materials verbally rather than physically interacting or handing something in to be read) delivery made completion of the sessions less motivating. Despite this caveat, the high completion rates over 12 weeks (3 baseline and 8 intervention sessions) suggest participating through either delivery modality was acceptable to participants. The themes from the research journal also support this conclusion; although some participants assigned to telehealth were initially apprehensive or even disappointed to be assigned to the telehealth condition, as sessions progressed the theme “different but not worse” as a description of videoconferencing-delivered sessions emerged from the research journal entries. These qualitative data revealing apprehension about telehealth could have interacted with the decision to withdraw made by the 2 participants initially assigned to telehealth, but who withdrew after the initial assessment and before the intervention. In order of theme of “different but not worse” to be created, an individual needed to first interact through telefhealth to discover that expectancies regarding apprehension of technology were unfounded.

Importantly, the results suggest participants’ goal performance improved across both treatment delivery modalities. Of the 15 goals set in this study, performance on only two goals (sleep set by Participant C, and concentration set by Participant D) did not improve by 2 or more points on the COPM. Participants C and D were both assigned to the telehealth group, so this raises the possibility that telehealth may reduce the efficacy of cognitive rehabilitation for older adults with SCI. It may also be the case that these goals are less amenable to cognitive rehabilitation. Improved sleep, in particular, is not a typical goal for cognitive rehabilitation, however, improving sleep and managing daytime sleepiness were both reported as goals set in Clare and colleagues’ [26] study (goal attainment was not reported goal by goal). In the sleep intervention literature more generally, CBTi is an effective treatment, demonstrates efficacy that is similar to pharmacological interventions with better long-term outcomes, and has been recommended as a standard treatment for insomnia [68]. Importantly, a full course of CBTi, which is typically between 6 and 8 sessions was not delivered here (Ms C participated in 5 sessions that focused on her goal to improve her sleep). Overall, the results of this study suggest that it is worthwhile to pursue adapting cognitive rehabilitation to telehealth videoconferencing. This is consistent with previous research that has explored remotely-delivered cognitive rehabilitation (ie, [37]) as well as remotely-delivered psychotherapy (ie, [14,23]).

The importance of establishing a strong therapeutic relationship was a major theme that emerged from the research journal. This aspect of cognitive rehabilitation has perhaps not been emphasized enough in the literature, and clinicians who are providing the intervention (whether or not they have been trained as psychotherapists) may benefit from attending to the research on the common factors of psychotherapy (see [69] for a recent summary of the common factors literature based on meta-analyses). This is not to suggest that the therapeutic relationship has been ignored in the cognitive rehabilitation literature, but to highlight the importance of not emphasizing technique (ie, errorless learning and spaced retrieval) at the expense of developing an alliance. One imagines that telehealth videoconferencing could impact developing an alliance, however, this was not noted in the research journal and psychotherapy noninferiority trials (ie, [23]), and other videoconferenced work [14] detail how the therapeutic relation can be established and maintained remotely. Future researchers might consider adding a formal measure of alliance to their protocols.

### Limitations

In carrying out this study, the researchers learned a number of things that may be helpful for future researchers. First, the researchers were surprised by how challenging it was to recruit research participants with MCI or early-stage AD. Those recruited and retained in the study were highly motivated and engaged, which is a self-selection bias. This recruitment challenge and the way in which participants were randomly assigned to the in-person or telehealth videoconferencing limits the conclusion the researchers can draw about delivering cognitive rehabilitation through videoconferencing to individuals with MCI or dementia due to AD (the 3 telehealth participants were individuals with subjective memory impairment). The researchers were also surprised to find that the majority of the participants in this study opted to participate without a support person. This was either because no support person was available (Ms C), because a support person was not interested in participating (Mr B and Ms E), or because it was decided that the support person was not needed (Ms D and Ms F). Only Ms A’s husband accompanied her to every session. This is noteworthy because Ms A was the only participant with a diagnosis of dementia due to AD. Previous research (ie, [8]) has recommended that a support person always be included in the intervention. The results of this study suggest that for individuals with SCI a support person is not necessary, but for individuals with dementia due to AD or MCI, the researchers continue to recommend a support person.

The researchers also came to reconsider the experimental design. The multiple baseline design was chosen to be able to infer that any improvements in COPM scores were due to cognitive rehabilitation interventions rather than common therapeutic factors such as establishing a positive relationship with the researcher delivering the treatment. As the study progressed, it became apparent that skills being taught for one goal carried over to other goals, and in some cases (Participant D in particular), participants spoke explicitly about generalizing strategies from a goal that was being trained to a goal that was not being trained. Of course, this is excellent for that individual, but it does limit the usefulness of within-person multiple baselines for cognitive rehabilitation. Therefore, this type of experimental design is not recommended, at least not for similar goals. Furthermore, in this study the researchers chose to rely on visual inspection to examine these data. This has the advantage of highlighting strong effects, which are more likely to be functionally relevant. The limitation of this approach is that subtle trends such as serial dependency are not readily observable using visual inspection, visual inspection is unreliable when effects are not large, and for these reasons, statistical methods of analyzing single-case data have been increasingly studied and used [60].

### Conclusions

The findings presented in this study support developing goal-oriented cognitive rehabilitation delivered both in-person and expanding the accessibility of this intervention by adapting it to videoconferencing. Further research is needed to replicate the results presented here. Additionally, this data does not fully explore the adaptation of cognitive rehabilitation to videoconferencing for individuals with cognitive impairments consistent with MCI or dementia due to AD. Given the increasing prevalence of cognitive impairment in late life in both urban and rural areas, interventions aimed at supporting the personally relevant functional goals of these individuals are clearly needed.

## Appendix

Multimedia Appendix 1 CONSORT‐EHEALTH checklist (V 1.6.1).

## Abbreviations

AD: Alzheimer disease
ADLs-Bristol: Bristol Activities of Daily Living Scale
CBTi: cognitive behavioral therapy for insomnia
CG: caregiver
COPM: Canadian Occupational Performance Measure 4th Edition
D-KEFS: Delis Kaplan Executive Function System
HADS: Hospital Anxiety and Depression Scale
MCI: mild cognitive impairment
MCID: minimum clinically important differences
MMSE: Mini-Mental State Exam
QoL-AD: Quality of Life in Alzheimer Disease
RBMT-III: Rivermead Behavioral Memory Test III
RCI: reliable change indices
SCI: subjective cognitive impairment
SED: standard error of the difference
SEM: standard error of measurement
TBI: traumatic brain injury
TEA: Test of Everyday Attention
WHOQOL-BREF: World Health Organization Quality of Life Assessment, short version
ZBI: Zarit Burden Inventory
